# The Nervous System

**Published:** 1849-04-01

**Authors:** 


Art. VII.—*A New Exposition of the Functions of the Nerves.* By James William Earle.

*The Brain the sole Centre of the Human Nervous System.* By Edwin Lee, Surgeon. 1849. (Read before the Royal Society, Edinburgh.)

Mr. Lee's paper is of recent publication. Mr. Earle's appeared several years ago. In both, the same thesis, the brain the sole centre of the human nervous system, is maintained. Mr. Earle has endeavoured to establish "a more correct and philosophical classification of the nerves," by bringing them within the precincts of "a system" analogous to that of "the vascular circulation." The brain, medulla, spinal cord, ganglia, and nerves, constitute the nervous system. As a "system," this is susceptible of certain subdivisions founded upon diversities of function. Both these works contain much that is suggestive, if not absolutely conclusive.

We give precedence to Mr. Earle's views, he having been first in the field. His preliminary chapter is devoted to the subject of irritability. The author starts by asserting, in opposition to the views of Haller, as accepted by Dr. W. Philip, and adopted by Dr. Carpenter, the following principles:—"There is no proof," he says, "that the contractility or excitability of the voluntary muscles, or that the action of the heart and arteries, is independent of the nervous system; or that nervous influence is independent of the brain or identical with galvanism." In this part of his essay, the author is not, perhaps, sufficiently perspicuous. The reader is expected to take rather spacious mental strides with him. His observations upon this portion of his subject are nevertheless acute. He handles rather summarily Dr. Philip's use of the term "sensorial power," as being too indefinitely employed. Mr. Earle considers volition and sensation as bearing an opposite relation to each other—the one being an active, the other a passive property, and each entirely independent of the other. He concludes the chapter by some remarks on the circumstances which attend the life and death of an acephalous fœtus: citing the observation of Mr. Lawrence, that "there is no recorded instance of a heart being found in a fœtus in whom no spinal marrow existed;" and by an inquiry into the reason why the heart of an animal continues to beat for a short time after the head is removed. But for his elucidation of these points we must refer to the author's text.

In the second chapter, the nature of nervous influence and the classification of the nerves come under consideration. "A something," he observes, "is constantly emanating from the brain, which has been called nervous influence." In support and illustration of his view, he adduces John Hunter's observations respecting the magnitude and number of the nerves bestowed upon the electric organs of fishes. He is at issue however with Hunter, in so far as that great physiologist regarded their electric power as the production of the nerves themselves; "because after the exhaustion of the nervous apparatus, it is necessary to let the animal rest for some hours before shocks can be reproduced; but by rest alone, he argues, it would remain in *statu quo,* unless some positive action had gone on during rest, and thinks that there is no difficulty in solving the question by attributing the reproduction of this fluid to a process, by means of which it may gradually accumulate in the nerves." Mr. Earle thus by a felicitous train of argument and illustration, glancing at the phenomena of luminous insects, brings the reader to consider a most important function of the brain, which he thinks ought to be henceforward distinguished by the term *secernent function.* Under this view it would be well to note in man and other animals, the proportion that those parts of the brain which govern large muscular and secernent nervous developments bear to those developments—an inquiry, the results of which would probably throw much light upon the question. He considers the secernent function to be never at rest for a single moment, and to be the action of the whole of the brain; while he believes, on the other hand, that volition and sensation belong only to certain parts of the brain. He next inquires into the structure of the nervous filaments, and endeavours to point out how these severally act. These points ascertained, should, he contends, determine their classification. Mr. Earle classifies them thus—*The first class* he forms of the olfactory, ophthalmic, and auditory nerves, which alone he terms cerebral nerves, these being the most simple in their character and functions. *His second class* comprehends every nerve by which muscles are subjected to the influence of volition, and includes those which belong to the anterior columns of the spinal cord. This division embraces the third, fourth, the anterior root of the fifth, the sixth, the portio dura of the seventh, and the ninth nerves. He considers all the medullary fibres below the first formation of the crura cerebri as comprised under the term anterior columns of the spinal cord, without regard to the circumstance of these fibres being encompassed by the pons Varolii, or separated by the *corpora olivaria. The third class* should comprehend every nerve belonging to the posterior columns, thus including the posterior root of the fifth, the glosso-pharyngeal, the pneumogastric, and spinal accessory nerves, all of which, with the exception of the last, transmit impressions from their extremities to the brain. By the term posterior columns is signified their whole extent as far as the termination of the restiform bodies in the posterior peduncles of the cerebellum. He invariably distinguishes the ganglions of the posterior nerves from those of the sympathetic, by the term *posterior ganglion*. Lastly, *the fourth class* should comprehend every nerve proceeding from the sympathetic ganglions. Every part to which these are distributed has a power of motion independently of the will. One well known fact, which tends to the support of this mode of classification, seems to have been either unrecognised or kept much in the back-ground; it is, that, nervous filaments, however intermingled, whether united with others near to or at a distance from the brain and spinal cord, never change their character. Motions are excited by irritating the posterior roots, but these are entirely dependent upon their connexion with the spinal cord, and upon the integrity of the anterior roots of the trunks into which they enter. In excitation of the posterior roots, the action upon muscles is not direct but reflex. The posterior roots constitute the channel through which the impression is conveyed; a motor impulse is excited in the spinal cord, which is propagated through the anterior roots to the periphery of the system. Mr. Earle has also distributed these classes into orders, "according to their natural arrangement, and the functions which they perform in combination." Of the first class (the cerebral nerves above named) he forms his *first order*. Of the nerves which come next to these in simplicity of function, he forms the *second order*, comprising those formed by the union of anterior and posterior filaments, or second and third classes, by which, bones, muscles, &c., and parts admitting of motion, and being under the control of the will, are supplied. The *third order* is formed of such as supply parts not under the control of the will, whose action is perpetual, neither taking nor needing rest, as the heart, arteries, intestines, and iris; such communication of nervous influence taking place through the union of posterior filaments, with filaments from the sympathetic ganglia. The *fourth order* consists of nerves formed by the union of anterior, posterior, and sympathetic filaments. Muscles thus supplied can support constant motion without fatigue, but differ from those instanced in the third order, by being always also obedient to the will. The nerves and function of the diaphragm exemplify this. Mr. Earle here notices some irregularities, as the union of sympathetic filaments with the phrenic, and of the portio dura with the fifth; again calling attention to the fact, that nerves have no action but at their extremities, and that thus there is no confusion of function, it being immaterial whether they proceed in company, or are joined just before their termination. It will be seen that the first order comprises single nerves; the second and third, couplets; the fourth, triplets of filaments. The posterior are common to all the combined nerves, all tissues requiring a constant stream of nervous power to maintain them in a healthy state. Cerebral influence is next considered, and a theory most ingeniously advocated, which we incline, with our gifted essayist, to think correct. A CIRCULATION OF NERVOUS POWER, as complete as that which is carried on by the arteries and veins, is ably traced out. Is not this view of the circulation of the nervous fluid strongly corroborative of the theory propounded by Mr. Alfred Smee in the preceding article? This circulation of the nervous system has complexities which Mr. Earle most lucidly explains. We regret much our want of space to do him justice. We are obliged to content ourselves with referring the reader to the author's own text, the study of which will amply repay him.

In Mr. Earle's third chapter he considers the functions of the second order of nerves, and combats the opinion of Dr. Philip, that the excitability of muscles is independent of the nervous system. There is some little obscurity in his mode of treating this subject. In the fourth chapter the functions of the third order of nerves are considered. His observations are chiefly confined to those of the heart and arteries. Here again he is at issue with Dr. Philip. Relying on the rule laid down by Sir Isaac Newton, that "sound philosophy teaches that activity in matter can never be inherent," he draws conclusions from the experiments made by Dr. Philip which are very different from those at which that gentleman himself arrives, maintaining that the brain is the source whence the influence of the nerves is derived.

The next portion of his work is divided into five sections. The influence of the nervous system upon secretion, the fluidity of the blood, the evolution of animal heat, and the motor and secernent functions of the animal economy, is the subject of comment. These sections display great force of argument, abound in original ideas and instructive suggestions, and supply much matter for reflection. We shall give some of these briefly. These are, his decisions that galvanism and nervous energy are not identical, inasmuch as the former exhausts in proportion as it excites nervous energy; that the fluidity of the blood is owing to its contact with living parts; that animal heat is mainly generated by nervous influence. There is also an inquiry into the mode in which, by means of nerves, cerebral influence is rendered available in maintaining the motor and secernent functions.

The fifth chapter treats of associations of nerves formed by the triple union of anterior, posterior, and sympathetic filaments; the power of will over the muscles, their supply being only exerted as occasion or convenience may require. The muscles of the face and the intercostals belong to this order,—their involuntary action is secured by branches from the sympathetic ganglia; they are subjected to the will by anterior filaments, in the same way as the diaphragm, and they have posterior filaments for the maintenance of a healthy state of structure. It is important that we should notice that Mr. Earle repudiates Sir Charles Bell's views respecting the respiratory tract, upon which that late distinguished physiologist based much of his reputation. He doubts the existence of the respiratory tract, and endeavours to establish that the so-called "respiratory nerves" have another origin. These are intricate points of controversy, upon which, had we enough of time and space at command, we should be glad to expatiate; but we must omit their consideration at present. The distribution of Mr. Earle's fourth order of nerves is extensive, including the third, fourth, fifth, sixth, portio dura of the seventh, glosso-pharyngeal, the primary branches of the pneumogastric, the ninth, and the anterior branches of all the spinal nerves except the fifth, sixth, and seventh cervical nerves. In the course of the wanderings of the various nervous filaments, none are found without their destinations. The nervous system is "a mighty maze, but not without a plan;" the paths which its filaments traverse are as regular and intelligible as those of the planets. Mr. Earle has ably contributed towards a solution of the difficulty.

We now turn to Mr. Lee's pamphlet. As will be seen by the title, Mr. Lee brings the reader to the same conclusion as that to which Mr. Earle conducted him. He regards the brain as the source of that one power which we commonly call nervous influence. He commences with remarks on the two great divisions of structure in the nervous system—the vascular grey and the fibrous white matter: the former being characterized as the generator, the latter as the conductor, of nervous power. Mr. Lee very properly objects to conclusions drawn from experiments on inferior animals, as applicable to the mammalia. In man especially, and in all the higher mammalia, the grey matter is mostly agglomerated in the brain. Where the brain is small, reactive effects may be produced for some time after its removal, but where it is large, such reactive effects either do not take place at all, or soon cease. After noticing the opinions of Bichat and Kolliker, who considered the sympathetic system and ganglia as the seat of special activity, he refers to pathological facts, and quotes the authority of Galen, Willis, Longet, Kaule, and Müller, who all regard the sympathetic system as an appendage to the cerebro-spinal. The latter, although he thinks that the moveable parts, the heart and bowels, are to a certain extent independent of the brain and spinal cord, seems compelled to allow that these organs must be regarded as the source of the functions of the sympathetic, "*if its power is not to be exhausted.*" Hence Mr. Lee infers "that the sympathetic is charged from the central parts of the nervous system, and that being once laden it retains its charge." "If, however, the brain and cord lose the capability of being the source of the nervous principle, exhaustion ensues, whence the debility and scarcely perceptible pulse at the close of acute diseases." "It is hence that we can account for the uninterrupted persistence of the organic functions during sleep." In noticing the theory of Dr. Marshall Hall respecting "the exclusive functions of the spinal marrow, and a peculiar system of excitor and motor nerves, of which it is the true axis," (we are here quoting Dr. M. Hall's words,) he supports his own and Mr. Earle's theory respecting the influence of the brain. Mr. Lee asserts that the influence of the brain is primarily exerted in these actions, although exerted in a circuitous manner. M. Duges considers, in accordance with Mr. Lee's theory, that the cerebral nerves are the chief centres of innervation, and that the spinal cord is rather intended to conduct than elaborate impressions and mental determinations. How much the brain has to do with development and growth, is to be inferred from the larger relative proportion of this mass in children. The nervous power retained in the spinal cord for the immediate action of the parts which it supplies, is soon exhausted upon its being cut off from the primary source of nervous energy. Müller has remarked, that "the existence of distinct paralysis of sensation and motion tends to prove that the sensitive and motor filaments pass up separately to the brain."

We quote largely from Mr. Lee, because we do not think his opinions capable of being expressed more lucidly than by himself: we take this course also, because we are compelled to restrict ourselves within the compass of an analysis rather than a full and elaborate critique. "It has been ascertained," he says, "that nerves do not terminate, as was formerly supposed, in the parts which they supply, but are reflected in loops; and that the filaments of which a nerve is composed, though bound together in the same sheath, yet pass separately into the spinal cord, and, according to some physiologists, (Valentin,) on to the brain, forming loopings also in the cerebral hemispheres, coming in contact in their passage along the cord with its grey matter; and this view appears to be corroborated by the phenomena of disease." "In proportion as nervous filaments pass through a greater quantity of grey matter, are their sensitive as well as reflex influences open to observation." There are some of his conclusions which we do not so readily follow, and which, in so far as we keep pace with him, fail to meet with our entire concurrence. The foregoing will present a concise, but tolerably faithful, analysis of Mr. Lee's general views of a very important and highly interesting subject.

After quoting Professor Cruveilhier, MM. Foville, Pinel, Grandchamp, Flourens, Cabanis, and others, in confirmation of his views of the dependence of the spinal cord on the brain, he adds, that "the same objections that have been made with reference to reflex action as depending solely on the cord, apply also to muscular tonicity." Finally, he concludes his interesting and suggestive essay by submitting to the reader the following deductions:—

I. That in man, and the higher mammalia, the brain is the sole centre of the nervous system, and the source of power.

II. That the grey matter of the spinal cord, and the ganglia of the sympathetic, are to be regarded as reservoirs of this power for the immediate actions of the parts supplied, which speedily become exhausted if communication with the brain be cut off; and also as modifiers of impressions transmitted from the periphery to the brain.

III. That the automatic and instinctive actions are not performed independently of the concurrence of the brain, though its influence is less direct and manifest than in the case of voluntary movements.

IV. That the brain is the organ principally implicated in several disorders, which have been too extensively referred to the spinal cord.

